# Transcriptomic profiles of age-related genes in female trachea and bronchus

**DOI:** 10.3389/fgene.2023.1120350

**Published:** 2023-03-08

**Authors:** Jia Liu, Haiyan Lu, Silu Hu, Faping Wang, Xiaoju Tang, Huajing Wan, Fengming Luo

**Affiliations:** ^1^ Clinical Research Center for Respiratory Diseases, Department of Pulmonary and Critical Care Medicine, West China Hospital, Sichuan University, Chengdu, China; ^2^ Laboratory of Pulmonary Immunology and Inflammation, Frontiers Science Center for Disease-Related Molecular Network, Department of Respiratory and Critical Care Medicine, West China Hospital, Sichuan University, Chengdu, China

**Keywords:** airway epithelium, trachea, bronchus, RNA sequencing, age

## Abstract

**Background:** Studies demonstrated that age-related cellular and functional changes of airway significantly contribute to the pathogenesis of many airway diseases. However, our understanding on the age-related molecular alterations of human airway remains inadequate.

**Methods:** Airway (trachea and bronchus) brushing specimens were collected from 14 healthy, female non-smokers with ages ranging from 20 to 60 years. Bulk RNA sequencing was performed on all the specimens (*n* = 28). Airway cell types and their relative proportions were estimated using CIBERSORTx. The cell type proportions were compared between the younger (age 20–40) and elder group (age 40–60) in the trachea and bronchus respectively. The linear association between cell type proportion and age was assessed using the Pearson correlation coefficient. Differentially expressed genes (DEGs) between the two age groups were identified using DESeq2. Three kinds of enrichment analysis of the age-related DEGs were performed, including Gene ontology (GO) enrichment, Kyoto Encyclopedia of Genes and Genomes (KEGG) pathway enrichment, and disease enrichment analysis.

**Results:** Sixteen and thirteen cell types were separately identified in tracheal and bronchial brushings, with the airway epithelial cells (including suprabasal, submucosal gland (SMG) goblet, serous, secretory, multiciliated, cycling.basal, basal cells) accounting for 85.1% in the trachea and 92.5% in the bronchus. The lymphatic cell and NK cells had a higher abundance ratio in the trachea, compared with the bronchus. The proportion of basal cells was negatively related to age both in the trachea and bronchus. Thirty-one and fifty-two age-related DEGs (*p* < 0.1) were identified in the trachea and bronchus, respectively. Among them, five common DEGs (CXCL2, CXCL8, TCIM, P4HA3, AQP10) were identified. Pathway enrichment analysis showed both tracheal and bronchial age-related DEGs were primarily involved in immune regulatory signaling pathways (TNF, NF-kappa B, IL-17 et al.). Disease enrichment analysis suggested that tracheal age-related DEGs significantly related to asthmatic pulmonary eosinophilia, and chronic airflow obstruction et al., and that bronchial age-related DEGs were enriched in airflow obstruction, bronchiectasis, pulmonary emphysema, and low respiratory tract infection et al.

**Conclusion:** We found the proportion of basal cells decreased with age in both the trachea and bronchus, suggesting a weakening of their self-renew ability with age. We identified transcriptomic signature genes associated with the early aging process of the human trachea and bronchus, and provided evidence to support that changes in their immune regulatory function may play critical roles in age-related airway diseases.

## 1 Introduction

Ageing is a process of time-dependent accumulation of cellular damage which results in a decline of airway function (Schneider et al., 2021). Emerging evidence demonstrated that age is not only a risk factor for airway diseases, but also significantly contributes to the pathogenesis and traits of many airway diseases (Nagasaki and Matsumoto, 2013; Occhipinti et al., 2017; Budde and Skloot, 2022). For example, 1) the prevalence of chronic pulmonary diseases, including chronic obstructive pulmonary disease (COPD), lung cancer and idiopathic pulmonary fibrosis (IPF) increase significantly with age (Authors Anonymous, 2000; Gao et al., 2020; CDC, 2021).For example, the prevalence of COPD in people aged over 40 was up to 13.6% (Fang et al., 2018), patients with IPF are often middle aged, usually over 40 years old (Authors Anonymous, 2000); 2) aged lung is more susceptible to SARs-CoV2 infection (Mueller et al., 2020; Chow et al., 2021); 3) ageing significantly influenced asthma in terms of symptoms, inflammatory features, prognosis and treatment response (Braman, 2017). Thus, in-depth characterizing the dynamics of the airway aging process will provide important clues to exploring the mechanism of aging-related airway diseases.

The luminal surface of the normal human airways, including the trachea and bronchi, are lined by epithelium which is composed of ciliated cells, club cells, basal cells and goblet cells et al. (Mercer et al., 1994). The airway epithelium is simultaneously exposed to both environmental and internal stimuli over a lifetime, and it gradually undergoes cellular and functional changes, including decreased number of broncho epithelial cells, decreased mucocilliary clearance and mucous production, decreased regeneration capacity (Wansleeben et al., 2014; Basil et al., 2020; Cho and Stout-Delgado, 2020). Recently, the advancement of the high-throughput RNA sequencing (RNA-seq) techniques enables us to build the cell and molecular atlas of human airway epithelium (Deprez et al., 2020). However, the transcriptional profiling of healthy human trachea and bronchus across the age range is not available. Herein, we collected tracheal and bronchial brushing samples from healthy females at different ages and characterized their transcriptomic signatures using bulk RNA-seq technology. We identified differentially expressed genes, analyzed their functions and diseases associations. This study established a valuable molecular map of airway ageing for future mechanistic investigations on airway ageing and age-related diseases.

## 2 Materials and methods

### 2.1 Subjects

The tracheal and bronchial brushings were acquired from healthy volunteers in a case-control and cohort study approved by the Ethics Committee of the West China Hospital, Sichuan University (approval number 2019-246). The inclusion criteria for enrolled participants were: 1) None currently existing respiratory symptoms, 2) A virtual normal chest imaging, 3) None smoking history, and 4) None self-reported occupational exposure to dust. We confined the exclusion criteria to subjects with a history of chronic respiratory diseases or a regular, long-time medication intake. Finally, fourteen female subjects were enrolled, with an age range from 20 to 60 years old. To compare differences in the gene expression of the lungs according to age, the subjects were classified into two groups based on their age: the younger group (20–40 years) and the elder group (over 40 years). We chose age 40 as a cutoff value to define the young and aged cohorts based on following reasons: 1) the prevalence of many respiratory diseases increase significantly after age 40, especially for age-related diseases, such as idiopathic pulmonary fibrosis (Authors Anonymous, 2000), and lung cancer (Gao et al., 2020) and COPD(8); 2) linear declines of lung function were observed around 40 in healthy populations (Janssens et al., 1999).

### 2.2 Sample acquisition

Tracheal and bronchial brushing samples were attained by flexible bronchoscopy (model BF-1TQ290, Olympus, Tokyo, Japan). The workflow of the flexible bronchoscopy was based on the British Thoracic Society guidelines (Du Rand et al., 2011). Subjects were required to fast for 4–6 h before the procedure. Then, intravenous lines were established for administration of propofol. Blood pressure, heart rate, respiratory rate, and pulse oxygen saturation, were continuously monitored by the anesthesiologist and nurse during the flexible bronchoscopy (iPM patient monitor, Shenzhen Mindray Bio-Medical Electronics Co., Ltd., Shenzhen, China). The cessation of bronchoscopy was triggered when the SpO2 dropped to <90%, or the BP dropped to <90 mmHg, or the HR dropped to <40 beats/min, or the RR dropped to 8 breaths/min. The tracheal and bronchial brushing samples were collected from the surface of annular cartilage and the surface of superior segment in right lower lobe, respectively. Samples were immediately put into liquid nitrogen for transportation and in a −80°C freezer for storage.

### 2.3 RNA extraction

Total RNA was extracted using the RNA Nano 6000 Assay Kit of the Bioanalyzer 2100 system (Agilent Technologies, CA, United States). RNA integrity number was used for evaluating the RNA integrity. RIN is over nine in all 28 samples. After assessing RNA integrity, a total amount of 1 μg RNA per sample was used as input material for the RNA library preparations.

### 2.4 Transcriptome sequencing

Briefly, mRNA was purified using poly-T oligo-attached magnetic beads. Fragmentation was carried out using divalent cations under elevated temperature in First Strand Synthesis Reaction Buffer(5X). First-strand cDNA was synthesized using random hexamer primer and M-MuLV Reverse Transcriptase (RNase H-). Second-strand cDNA synthesis was subsequently performed using DNA Polymerase I and RNase H. Remaining overhangs were converted into blunt ends *via* exonuclease/polymerase activities. After adenylation of 3′ ends of DNA fragments, Adaptor with hairpin loop structure was ligated to prepare for hybridization. To select cDNA fragments of preferentially 370–420 bp in length, the library fragments were purified with the AMPure XP system (Beckman Coulter, Beverly, United States). Then PCR was performed with Phusion High-Fidelity DNA polymerase, Universal PCR primers, and Index (X) Primer. At last, PCR products were purified (AMPure XP system). NEBNext^®^ Ultra™ RNA Library Prep Kit for Illumina was used for library construction. After the libraries were constructed, the libraries were initially quantified using a Qubit 2.0 Fluorometer and diluted to 1.5 ng/ul, followed by the detection of the insert size of the libraries using an Agilent 2100 bioanalyzer, and after the insert size met the expectations, the effective concentration of the libraries was accurately quantified by qRT-PCR (effective library concentration above 2 nM) to ensure the quality of the library. The library preparations were sequenced on an Illumina Novaseq platform and 150 bp paired-end reads were generated.

### 2.5 Genes expression analysis

Clean data were obtained by removing reads containing adapter, reads containing ploy-N and low-quality reads from raw data. At the same time, Q30 and GC content of the clean data were calculated. All the downstream analyses were based on clean data with high quality. Hisat2 (v2.0.5) was used/conducted to map clean reads to the reference genome (human genome GRCh38) (Kim et al., 2019). Then, featureCounts were performed to generate genes’ count matrix for those 28 samples, and fragments per kilobase of exon model per million mapped fragments (FPKM) values were calculated.

### 2.6 Profiling the cell type composition

The cell proportion of tracheal and bronchial brushings were calculated with CIBERSORTx (https://cibersortx.stanford.edu) (Newman et al., 2019). The single cell gene expression matrix generated by a previous study (Deprez et al., 2020) was accessed through web tool (https://www.genomique.eu/cellbrowser/HCA/). File titled “exprMatrix.tsv—Gene x Cell normalized expression table” was downloaded as the single cell gene matrix. The matrix of FPKM values for all samples was uploaded to the CIBERSORTx website as the Mixture file. The matrix from single cell atlas which includes trachea and intermediate bronchus samples in the healthy airway was selected as the Signature gene file (Deprez et al., 2020) (EGAD00001005714), while other parameters retained the default setting.

### 2.7 Differentially expressed gene screening

DESeq2 package (version 1.32.0) in R software (R version 4.1.0: https://www.r-project.org/) was adopted to explore the differential gene expression in two conditions (seven biological replicates in the younger and elder group) respectively for the trachea and bronchus (Love et al., 2014). DESeq2 provides statistical routines for determining differential expression in digital gene expression data using a model based on the negative binomial distribution. The resulting *p*-values were adjusted using Benjamini and Hochberg’s approach for controlling the false discovery rate. Age-related genes were screened with *p*-value < 0.1, absolute of log2FoldChange >0, and used for enrichment analysis. Volcano pictures of genes were constructed using the ggplot2 package (ggplot2, 2016).

### 2.8 Senescent phenotype in the trachea and bronchus

Aging-related genes obtained from The Aging Atlas (https://ngdc.cncb.ac.cn/aging/index) (Aging Atlas Consortium, 2021). The normalized expression of aging-related genes was selected from the FPKM matrix of the tracheal and bronchial brushings. The relationship between aging-related genes and age was evaluated through the Pearson correlation coefficient. The different expression of aging-related genes was explored in the younger and elder group, both in the trachea and bronchus.

### 2.9 Enrichment analysis

GO and KEGG pathway enrichment analysis of age-related genes was both implemented by the clusterProfiler R package (Yu et al., 2012). GO enrichment analysis at the biological process (BP) level was explored. Otherwise, disease enrichment was performed based on DisGeNET (version 7.0 at https://www.disgenet.org/) platform to explore the relationship between age-related genes and diseases (Piñero et al., 2017; Piñero et al., 2020; Piñero et al., 2021).

### 2.10 Statistical analysis

Student’s *t*-test was utilized for the comparison of two sample groups through GraphPad Prism (version 6, San Diego, CA) software. Statistical significance of the correlation of cell proportion and age was assessed by the Pearson correlation coefficient. Multiple hypotheses of using Benjamini–Hochberg method were done appropriately. Differences were considered statistically significant when *p* < 0.05. Values are means ± SEM.

## 3 Results

### 3.1 Bulk RNA-seq of tracheal and bronchial brushings

**FIGURE 1:**
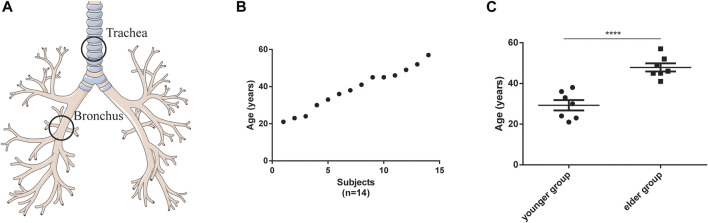
Characteristics of tracheal and bronchial brushings. **(A)** Schematic of brushing sample collection. **(B)** age distribution of the participants (*n* = 14). **(C)** Age difference between the younger (*n* = 7) and elder (*n* = 7) groups. ****p < 0.001.

Based on the inclusion and exclusion criteria listed in Materials and Methods, 14 healthy female participants were enrolled (detailed demographic information was listed in Supplementary Table S1). Their tracheal (*n* = 14) and bronchial (*n* = 14) brushing samples were collected by bronchoscopy (Figure 1A). The age of these participants was evenly distributed between 20 and 60 years (Figure 1B,C). Bulk RNA sequence was performed. For the 14 tracheal brushings, we yielded 90.47 Gb clean data after quality control, and approximately 95.81 percent were aligned to the h38 genome (Supplementary Table S2). For the 14 bronchial brushings, 91.12 Gb clean data was generated and their average h38 genome map ratio was 95.85 percent (Supplementary Table S2). Bulk tissue gene expression profiles for the trachea and bronchus were generated. After the quality filter (counts > 10), 26,884 genes were identified to be expressed. Those bulk tissue gene expression profiles were used for further assessment of cell composition, age-related changes of cell type proportion, age-related gene expressions, signaling pathway and disease enrichment analysis.

### 3.2 Age-related cell type composition changes in the airway

**FIGURE 2:**
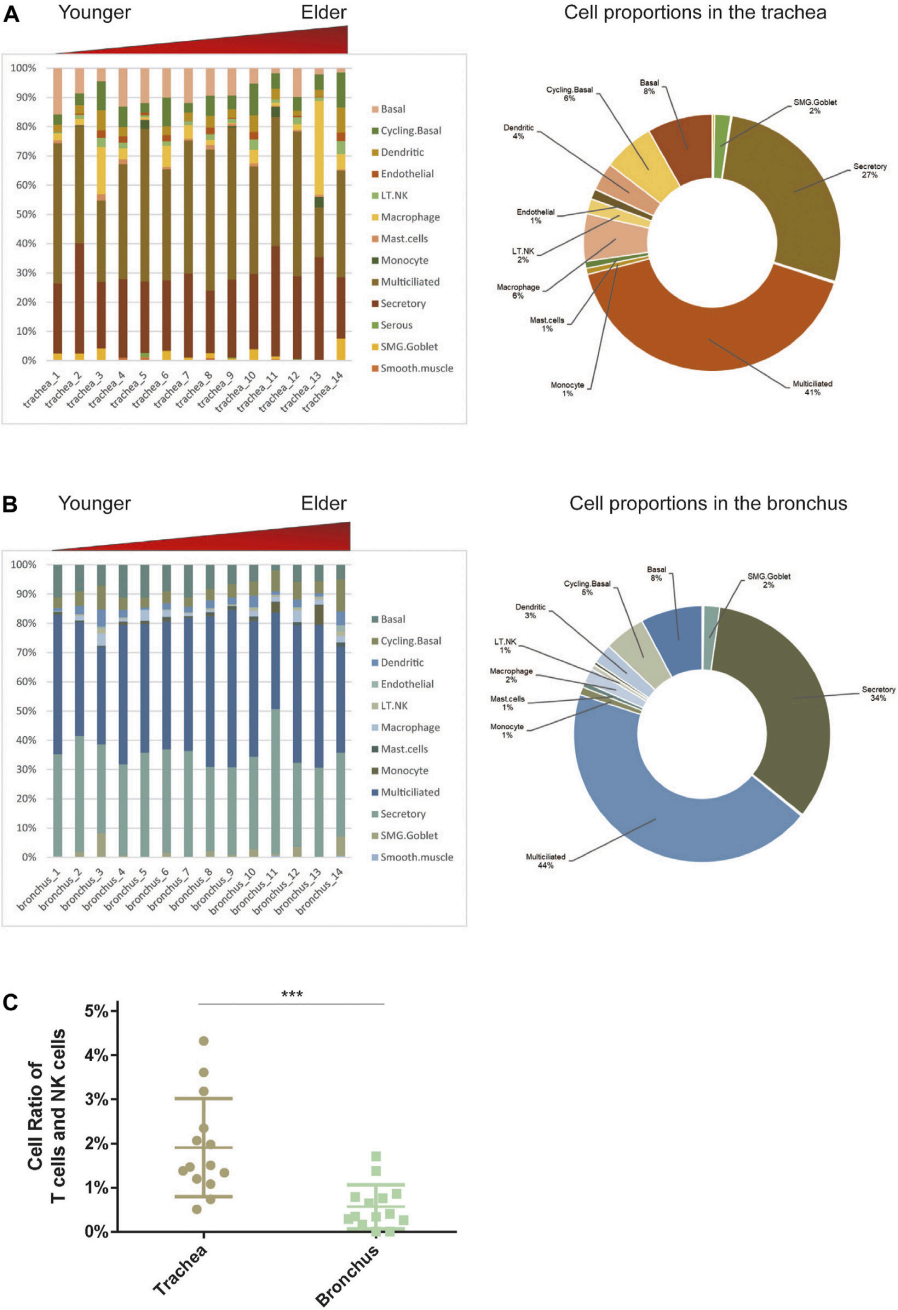
Estimate cell compositions in airway brushings using CIBERSORTx. **(A)** Trachea. **(B)** Bronchus. Notes: Cell type proportion in each sample (left), average cell type proportion (right). AT1 for type I pneumocytes; AT2 for type II pneumocytes; LT.NK for T lymphocytes and natural killer (NK) cells; PNEC for pulmonary neuroendocrine cells; SMG.Goblet for submucosal gland goblet. **(C)** The different cell ratios of T cells and NK cells in the trachea and bronchus. ***p < 0.01.

**FIGURE 3:**
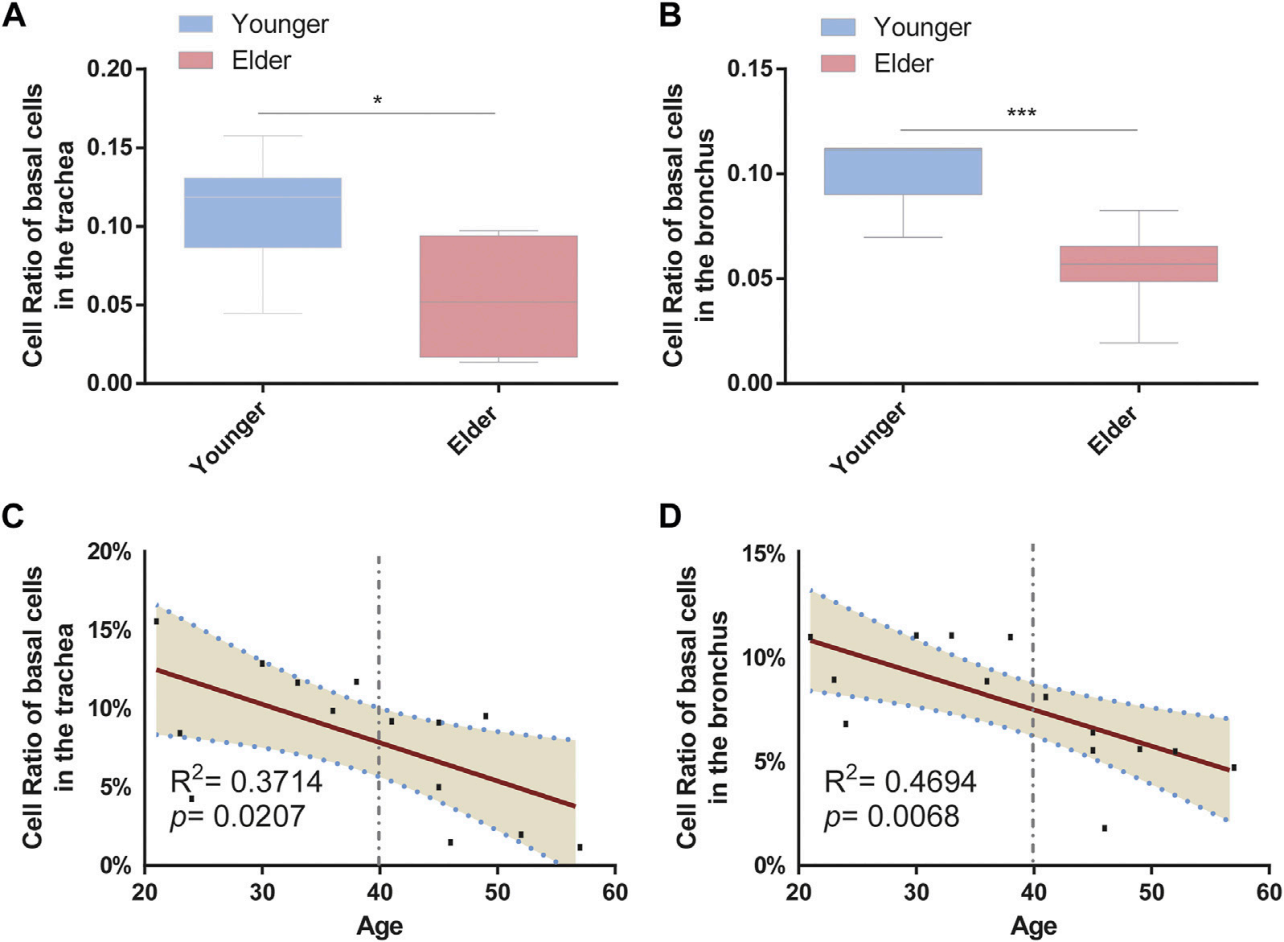
Proportion of basal cells decreased with age in the airway. **(A)** Proportion of basal cells between the younger and elder groups in the trachea. *p < 0.05. **(B)** Proportion of basal cells between the younger and elder groups in the bronchus. ***p < 0.001. **(C)** Pearson correlation analysis about the proportion of basal cells and age in the trachea. Pearson R and *p* are shown. The broken line represents the 95% confidence interval (CI). **(D)** Pearson correlation analysis about the proportion of basal cells and age in the bronchus. Pearson R and *p* are shown. The broken line represents the 95% confidence interval (CI).

To determine the cell-type composition of the trachea and bronchus, we used a publicly available single-cell sequencing dataset (Deprez et al., 2020) to construct an airway signature matrix and enumerate the cell composition from bulk gene expression profiles using CIBERSORTx (https://cibersortx.stanford.edu) (Newman et al., 2019). 16 and 13 types of cells were respectively identified in tracheal and bronchial brushings (Figure 2A). Among those cells, epithelial cells (suprabasal, SMG.goblet, serous, secretory, multiciliated, cycling. basal, and basal cells) were the dominating cell types, accounting for 85.1% in the trachea and 92.5% in the bronchus (Figure 2B). Other identified cell types include endothelial cells (trachea 1.2% ± 0.3%, bronchus 0.4% ± 0.1%), pericyte cells (trachea 0.1% ± 0.1%, bronchus 0.0% ± 0.0%), smooth muscle cells (trachea 0.3% ± 0.1%, bronchus 0.2% ± 0.1%), macrophage (trachea 6.0% ± 2.3%, bronchus 1.9% ± 0.4%), T lymphocytes and natural killer cells (trachea 1.9% ± 0.3%, bronchus 0.6% ± 0.1%), and dendritic cell (trachea 3.5% ± 0.6%, bronchus 2.5% ± 0.4%). The serous cells (trachea 0.2% ± 0.1%), pericytes (trachea 0.1% ± 0.1%), and deuterosomal cells (trachea 0.1% ± 0.1%) were identified in the tracheal brushings, but not the bronchial brushings (Supplementary Table S3). The proportion of T cells and NK cells was significantly higher in the trachea than in the bronchus (10.0% ± 0.6% vs. 5.5% ± 0.7%, *p* = 0.0006) (Figure 2C). In order to characterize the age-related changes of cell composition in the trachea and bronchus, we compared the cell type proportion between the younger group and elder group, and evaluated the changes of cell type proportions with age using Pearson correlation test. The proportion of basal cells was significantly lower in the elder group in both trachea (elder group 5.5% ± 1.5% vs. younger group 10.8% ± 1.4%, *p* = 0.021) (Figure 3A) and bronchus (elder group 5.5% ± 0.7% vs. younger group 10.0% ± 0.6%, *p* = 0.0006) (Figure 3B). And a negative correlation between the basal cell proportion and age were identified in both trachea (*R* ^2^ = 0.3714, *p* = 0.0207) (Figure 3C) and bronchus (*R* ^2^ = 0.4694, *p* = 0.0068) (Figure 3D). To validate our findings on age related basal cell changes, we downloaded whole lung single cell RNA-seq data from an online dataset (GSE136831), which were generated from a IPF study (Adams et al., 2020; Zhou et al., 2022a). RNA-seq data of 17 healthy individuals with age range from 20 to 80 (Supplementary Table S1) were used to assess the changes of basal cell proportion with age. Consistent with our findings, a negative correlation between proportions of basal cells with age were identified (Supplementary Figure S1).

### 3.3 Characterization of age-related gene expression in the airway

To validate the senescence phenotype of our bulk tissue gene expression profiles, we investigated the alterations of aging-related genes in the trachea and bronchus. 500 aging-related genes from 10 functional categories were acquired through The Aging Atlas (https://ngdc.cncb.ac.cn/aging/index) (Aging Atlas Consortium, 2021) and analyzed, including, 1) altered intercellular communication, 2) cellular senescence, 3) deregulated nutrient sensing, 4) epigenetic alterations, 5) genomic instability, 6) loss of proteostasis, 7) mitochondrial dysfunction, 8) NF-kappaB related gene, 9) senescence-associated secretory phenotype, 10) others. 443 aging-related genes were found to be expressed in the trachea and bronchus. In the trachea, 19 genes were found to have a different expression between the two age groups, which mainly functioned in senescence-associated secretory phenotype, NF-kappaB related gene, and genomic instability. Of the 19 aging-related genes, a correlation of expression level with age was identified in 12 genes, including *MIX1* (*R* ^2^ = 0.5689, *p* = 0.0018) and *CDC42* (*R* ^2^ = 0.5577, *p* = 0.0021) (Supplementary Figure S2). In the bronchus, 25 genes were found to have a different expression between the two age groups, which functioned in senescence-associated secretory phenotype, genomic instability, loss of proteostasis, and altered intercellular communication. Of the 25 genes, a correlation of expression level with age was identified in 13 genes in the bronchus, including *GCLC* (*R* ^2^ = 0.6645, *p* = 0.0004), *MMP2* (*R* ^2^ = 0.5007, *p* = 0.0046) (Supplementary Figure S3). Thus, the bulk tissue gene expression profiles were capable to reflect the early aging process of the human airway.

**FIGURE 4:**
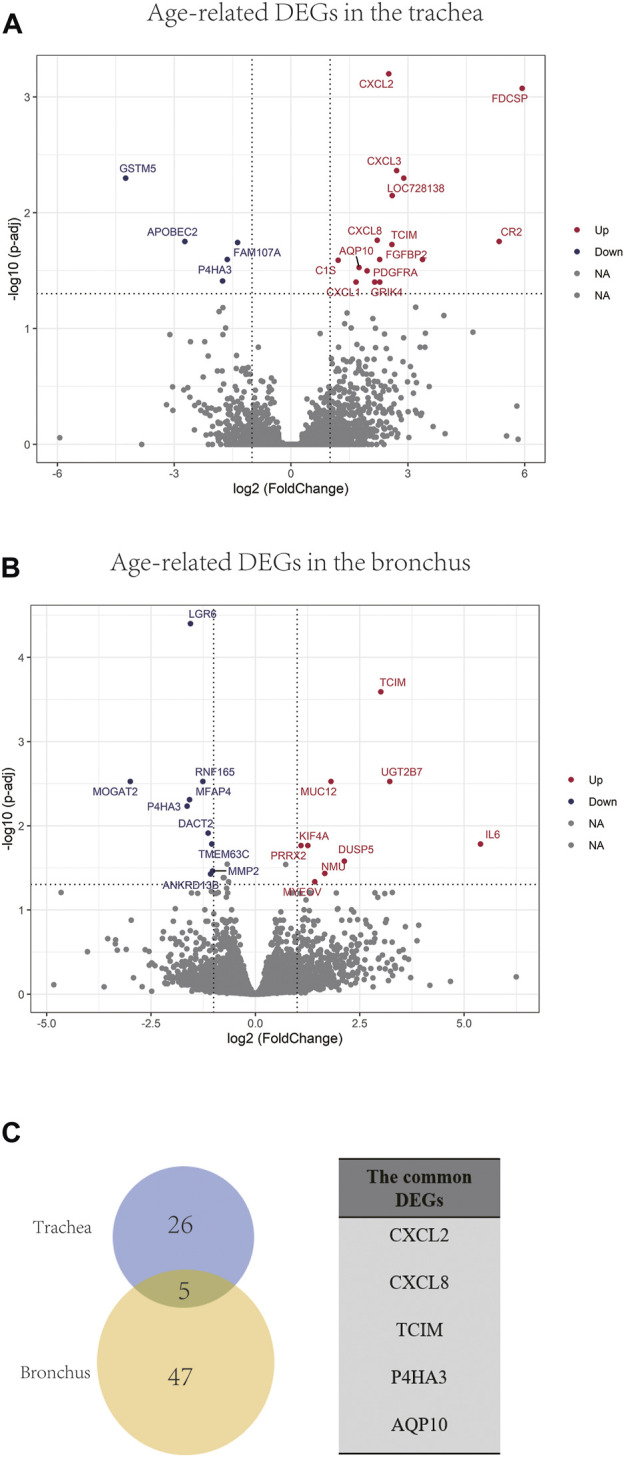
Age-related DEGs in the trachea and bronchus. **(A)** Volcano plot of age-related genes in the trachea. Genes with *p.adj* < 0.05 and at least 2-foldchange are labeled with gene symbols. **(B)** Volcano plot of age-related genes in the bronchus. Genes with *p.adj* < 0.05 and at least 2-foldchange are labeled with gene symbols. **(C)** Intersection of DEGs list in the trachea and bronchus and the list of common age-related genes in the trachea and bronchus.

In order to identify differentially expressed genes (DEGs) between the younger and the elder age groups, bulk RNA sequencing data of trachea and bronchus were analyzed using DESeq2 (Love et al., 2014). 31 tracheal DEGs and 52 bronchial DEGs were identified (Figures 4A, B). Among them, *follicular dendritic cell secreted protein* (*FDCSP*) was the most upregulated age-related DEGs in the trachea, and *interleukin 6* (*IL-6*) was the most upregulated age-related DEGs in the bronchus. Five common DEGs were found, including *CXCL2, CXCL8, TCIM, P4HA3, AQP10* (Figure 4C).

**FIGURE 5:**
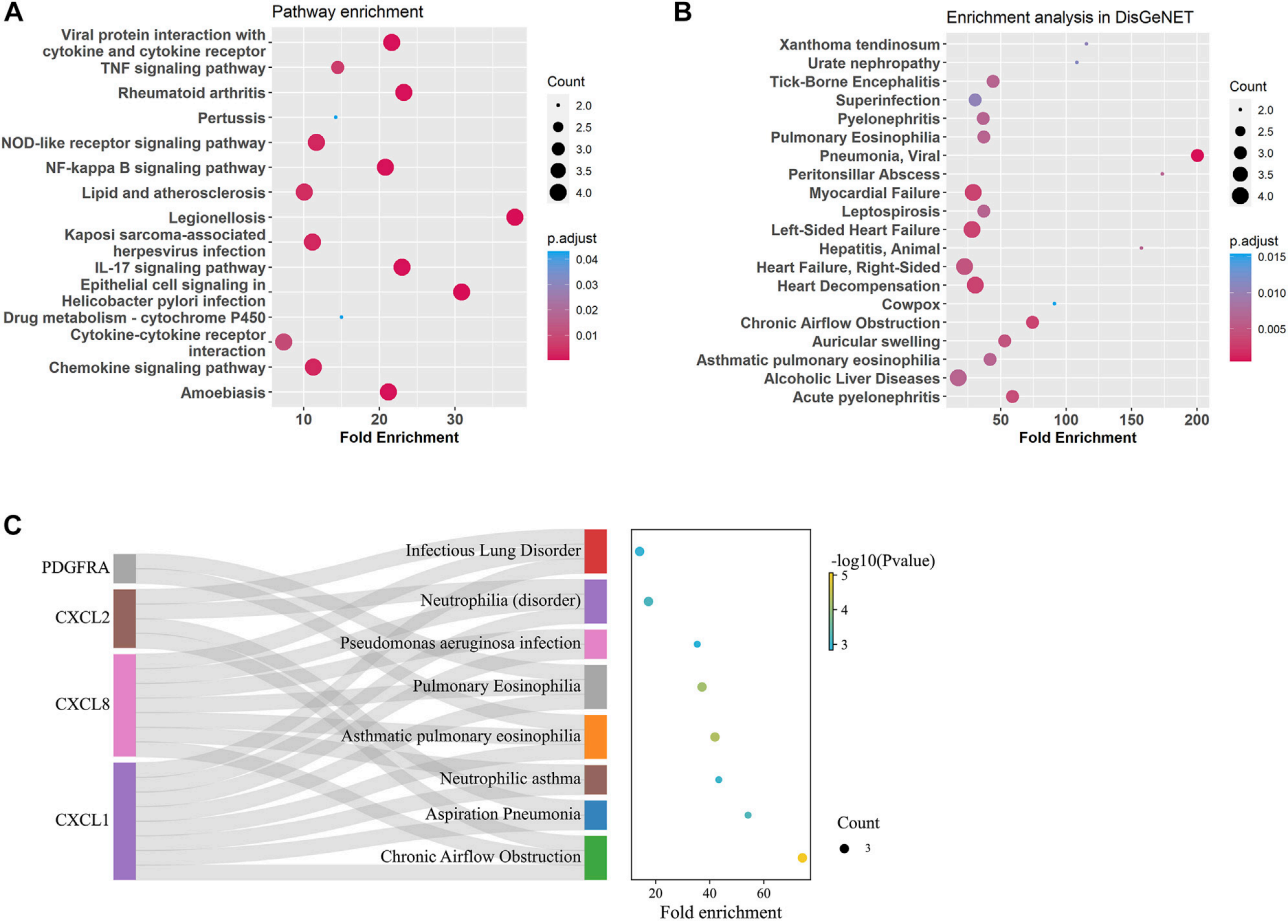
Enrichment analysis for age-related DEGs in the trachea. **(A)** Summary of KEGG enrichment in the trachea. **(B)** Summary of DisGeNET enrichment in the trachea. **(C)** Sankey plot showed relation between age related-DEGs and significant enriched (*p* adjust < 0.5) pulmonary diseases.

Gene enrichment analysis using tracheal DEGs overlapping with gene ontologies demonstrated that genes involved in response to chemokine, neutrophil and granulocyte migration, cellular response to molecule of bacterial origin, cell chemotaxis, and antimicrobial humoral immune response mediated by antimicrobial peptide were significantly enriched (supplementary figure S4). Gene enrichment analysis using tracheal DEGs overlapping with pathways from the Kyoto Encyclopedia of Genes and Genomes (KEGG) showed genes involved in TNF, IL-17, NOD-like, NF-kappa B, and chemokine signaling pathways were significantly enriched (Figure 5A). In order to test whether these age-related signature genes were associated with human diseases, DisGeNET(v7.0) database was used (Piñero et al., 2017; Piñero et al., 2020; Piñero et al., 2021). We found that age-related signature genes identified in the trachea were significantly associated with respiratory diseases, such as pulmonary eosinophilia, chronic airflow obstruction, and infectious lung disorder (Figures 5B, C).

**FIGURE 6:**
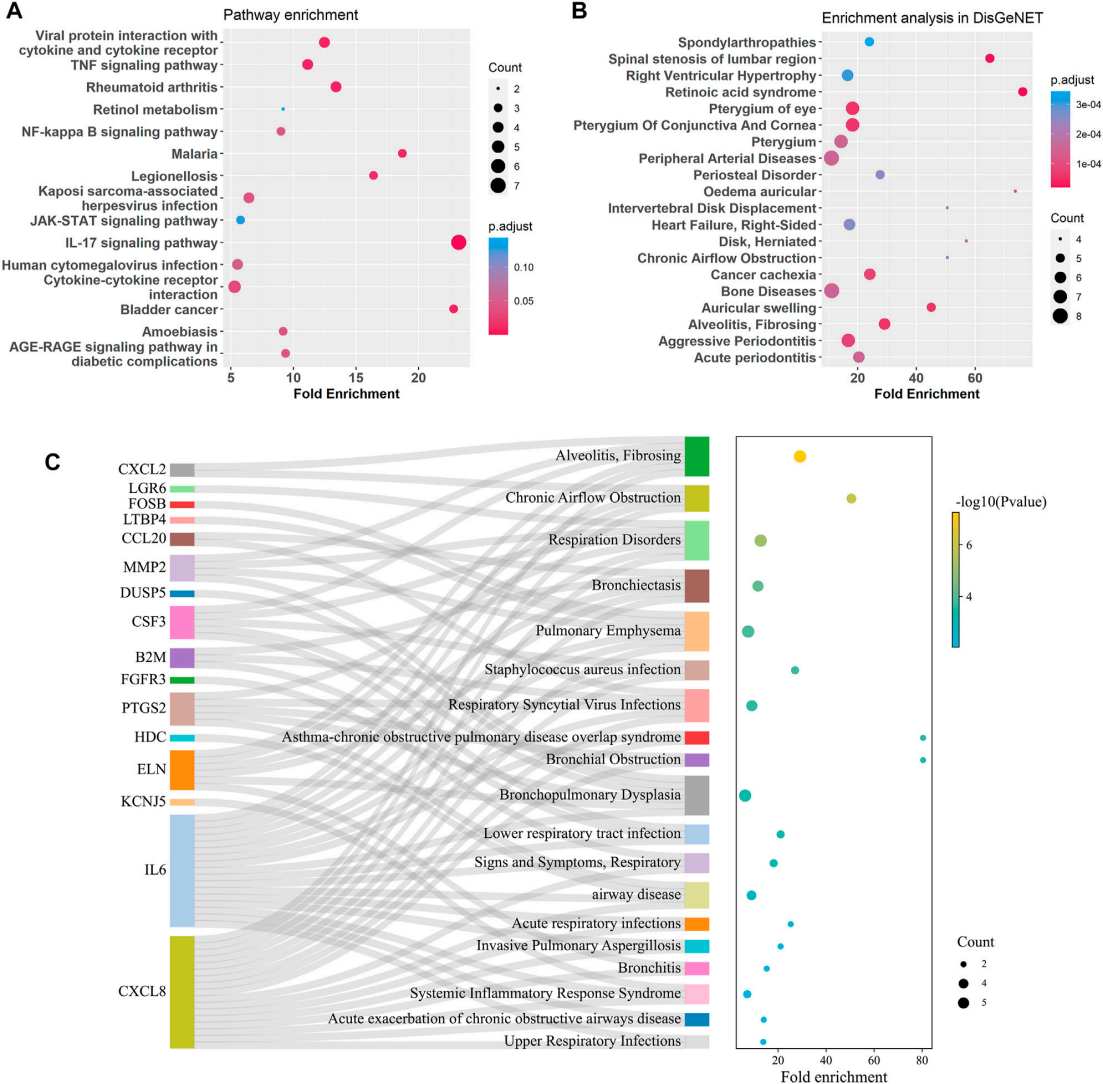
Enrichment analysis for age-related DEGs in the bronchus. **(A)** Summary of KEGG enrichment in the bronchus. **(B)** Summary of DisGeNET enrichment in the bronchus. **(C)** Sankey plot showed relation between age related-DEGs and significant enriched (*p* adjust < 0.5) pulmonary diseases.

GO enrichment analysis of the bronchial DEGs showed the top enriched biological processes were regulation of cell development, negative regulation of cell projection organization, and neurogenesis et al. (Supplementary Figure S5). KEGG pathway enrichment analysis of the bronchial DEGs revealed that IL-17, TNF, NF-kappa B, and JAK-STAT signaling pathways and viral protein interaction with cytokine and cytokine receptor were significantly enriched (Figure 6A). Disease enrichment analysis showed bronchial age-related DEGs were associated with respiratory diseases like alveolitis fibrosing, bronchiolitis, bronchial obstruction, bronchopulmonary dysplasia, and so on (Figures 6B, C).

## 4 Discussion

Although age-related gene expressions were explored in the lungs of mice and primates (Angelidis et al., 2019; Lee et al., 2021; Ma et al., 2021), our current study represents the first evaluation of age-related gene expression of healthy human airways using airway brushing samples. We identified transcriptomic signature genes associated with the early aging process of the human trachea and bronchus, and provided evidence to support that changes in their immune regulatory function may play critical roles in age-related airway diseases.

An airway single-cell sequencing study demonstrated that the human airway was composed of 89.1% epithelial cells, 6.2% immune cells, and 4.7% stromal cells (Deprez et al., 2020). Similar results were derived from our bulk RNA sequencing analysis using machine learning tools—CIBERSORTx, indicating bulk RNA sequencing data is also a reliable resource to assess the airway cell type composition. Basal cells, the airway epithelial stem cells, are required for airway maintenance and repair (Rock et al., 2010; Parekh et al., 2020; Schneider et al., 2021). We identified that basal cell proportion of the human trachea and bronchus were linearly decreased with age, suggesting a weakening of airway regeneration capability with age. Decreased basal cell and compromised airway regeneration capability were commonly observed in many airway diseases, including COPD (Stolz et al., 2022), further understanding of the molecular mechanism associated with airway basal stem cell aging will provide a framework to support stem cell therapy for airway diseases.

Chronic-low-grade inflammation were readily observed in the trachea of aged mouse (Wansleeben et al., 2014), and expression changes of genes involved in inflamm-aging and imbalance of immune activity were identified in the aged lungs of primate and mouse (Angelidis et al., 2019; Ma et al., 2021). Given the existence of species difference, whether these age-related changes were identical in human airway was unknown. Our study provided evidence to support that human tracheal and bronchial age-related DEGs were highly enriched in immune modulation and inflamm-aging. Five common age-related DEGs were identified in both trachea and bronchus. *CXCL2* and *CXCL8*, encode proteins as ligands of chemokine C-X-C chemokine receptor 2 (CXCR2), a powerful neutrophil chemotactic factor. The CXCLs/CXCR2 axis plays a vital role in apoptosis, epithelial-to-mesenchymal transition, and cell proliferation in lung cancer (Cheng et al., 2021), and unresolving neutrophilic airway inflammation of patients with COPD and severe asthma (Uddin et al., 2010; Simpson et al., 2013; Uddin et al., 2019). Knock-down of CXCR2 alleviates replicative and oncogene-induced senescence in human primary cells (Acosta et al., 2008). *TCIM* encodes a protein that functions as a positive regulator of the Wnt/beta-catenin signaling pathway and Notch pathway (Zhu et al., 2015), and found to promote the development of lung cancer (Su et al., 2013). The expression level of T*CIM* was significantly changed in COVID-19 infected lung and fibrotic lung (Evans et al., 2016; Nunnari et al., 2020). *P4HA3* encodes a protein as a key enzyme in collagen synthesis, facilitating growth and metastasis of multiple cancers (Song et al., 2018; Long et al., 2019; Gu et al., 2020; Zhou et al., 2022b). *AQP10* functions as water-permeable channels in the epithelia. *FDCSP* is the most increased gene identified in trachea, and it is reported to function as a B cell regulator. FDC-SP-deficient mice show significantly increased IgA levels in BALF (Hou et al., 2014). In lung squamous cell carcinoma, the up-regulated FDCSP was strongly enriched for the positive regulation of immune processes and inflammatory responses (Song et al., 2020). Interleukin-6 (IL-6) is the most increased gene in bronchial epithelium in the older adult group. IL-6 was a trigger of multiple inflammatory signaling pathways such as NF-kappa B and JAK/STAT, and reported to play essential roles in inflammatory response, immunity, and angiogenesis (Muraguchi et al., 1988; Hurst et al., 2001; Park et al., 2004; Martinez et al., 2009; Hunter and Jones, 2015; Rose-John, 2017; Murakami et al., 2019; Hirano, 2021). In addition to the genes listed in the aging atlas, we newly identified several genes *TCIM*, *FDCSP*, and *P4HA3* expressed differently during airway ageing, whether they play critical roles in airway aging and how they contribute to pathogenesis of airway diseases are worth to be further explored.

Airway diseases, such as COPD and asthma, remain leading causes of disability and death globally. Their prevalence increases significantly with age (GBD Chronic Respiratory Disease Collaborators, 2020). Profiling the age related gene expression in human airway and idenfying the association with diseases will direct us to explore the underlying molecular mechanism of the age-related airway diseases. Our disease association analysis demonstrated that the tracheal age-related DEGs were enriched in pulmonary eosinophilia, chronic airflow obstruction and infectious lung disorder et al., and the bronchial age-related DEGs were enriched in alveolitis fibrosing, bronchiolitis, bronchial obstruction, bronchopulmonary dysplasia, and so on. These findings support the concept that molecular changes associated with airway ageing significantly contribute to the pathogenesis of airway diseases. Further mechanistic researches focusing on molecules and signaling pathways changed significantly with age will help us to understand pathogenesis of airway diseases.

To avoids the confounding effects of smoking status and occupational exposure on gene expression, only 14 healthy female volunteers were qualified and enrolled in this study. Thus, limitations exist: first, RNA sequencing data from male are not available. Whether there is any difference of age-related gene expression between male and female cannot be explored. Second, the age range of the samples were from 20 years to 60 years old. Given the global average life expectancy of human is 72.9 years (Department of Economic and Social Affairs Population Division US, 2022), only early aging effects on gene expression can be detected in the current study. Third, samples for each age-group are limited, the gene expression heterogeneity caused by different individuals cannot be strongly avoid.

## Data Availability

The data presented in the study are deposited in the NGDC (https://ngdc.cncb.ac.cn/?lang=en), accession number PRJCA014015.
