# Supplementary material for: Transcriptomic profiles of age-related genes in female trachea and bronchus
Source: Front Genet. 2023 Mar 8;14:1120350. doi: 10.3389/fgene.2023.1120350 (PMC10031059; doi:10.3389/fgene.2023.1120350)
Supplement: Supplementary file 1 [file DataSheet1.pdf]

Supplementary Material

Supplementary Materials:

Supplementary Figure S1. Proportion of basal cells decreased with age in the 17 healthy lungs from GSE136831. GSE136831 is a dataset about IPF, we download the single cell RNA-seq data of 8 female and 9 male healthy lungs, and calculated the fraction of basal cells in each sample. Pearson correlation analysis about the proportion of basal cells and age in 17 healthy lungs from GSE136831. Pearson R and p are shown. The broken line represents the 95% confidence interval.

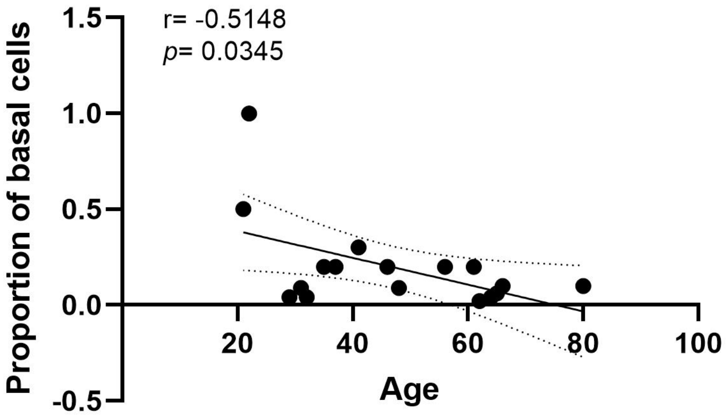

Supplementary Figure S2. Alterations of 19 aging-related genes with age in the trachea. (A) *CLCL8*, *CXCL1,* and *CXCL2* from senescence-associated secretory phenotype. (B) *TP63*, *CBX7*, and *RF4* from genomic instability. (C) *MXI1*, *CDC42*, and *SST* from others. (D) PTGS2, and PDFRA from altered intercellular communication. (E)

CTGF from stem cell exhaustion. (F) BIRC3, CARD10, TNFSF14, and BCL10 from NF-kappaB related gene. (G) FOS, and NRG1 from cellular senescence. (H) HTRA2 from loss of proteostasis.

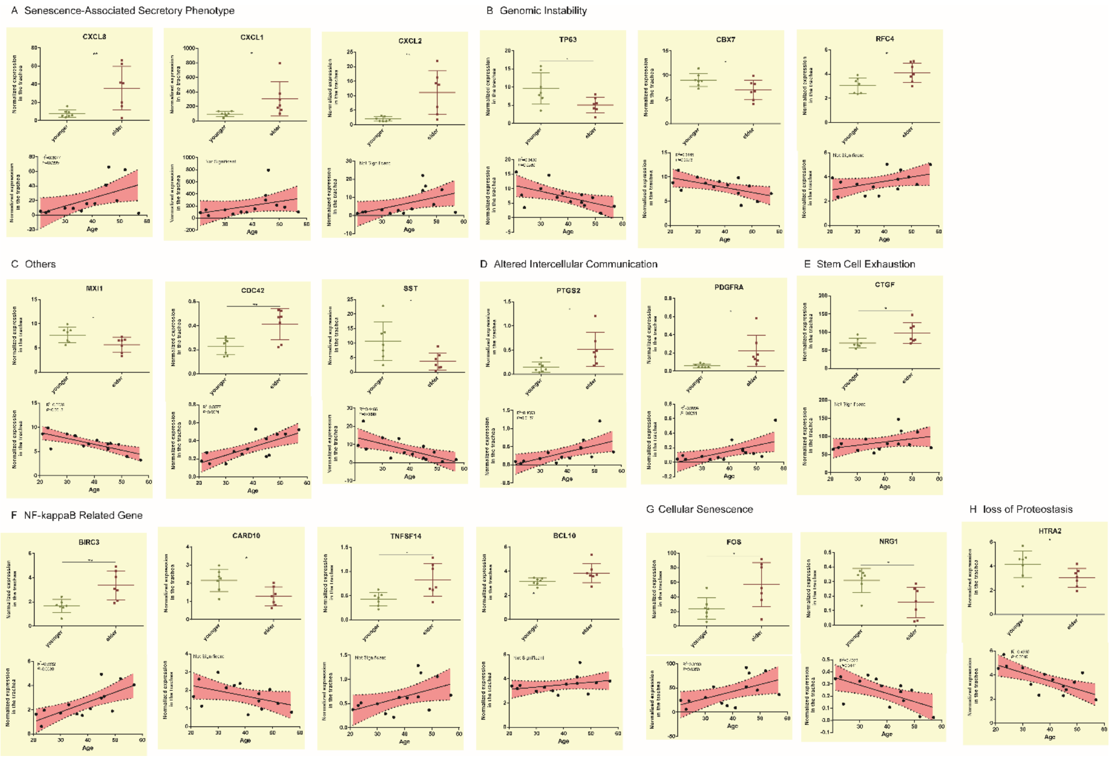

Supplementary Figure S3. Alterations of 25 aging-related genes with age in the bronchus. (A) *GCLC, DBN1,* and *HSPA8* from loss of proteostasis. (B) *HOXC4*, *TP63*, and *CBX7* from genomic instability. (C) *ELN*, *PRKACB*, and *PDGFRA* from altered intercellular communication. (D) MMP2, AXL, ARGE, CXCL8, CXCL2, and WNT2 from senescence associated secretory phenotype. (E) TSC2 and FOXO4 from deregulated nutrient sensing. (F)

TCF3 and INSR from cellular senescence. (G) PLCG1 and BCL10 from NF-kappaB related genes. (H) FGFR3, CEPA, ULK1, and NCOR2 from others.

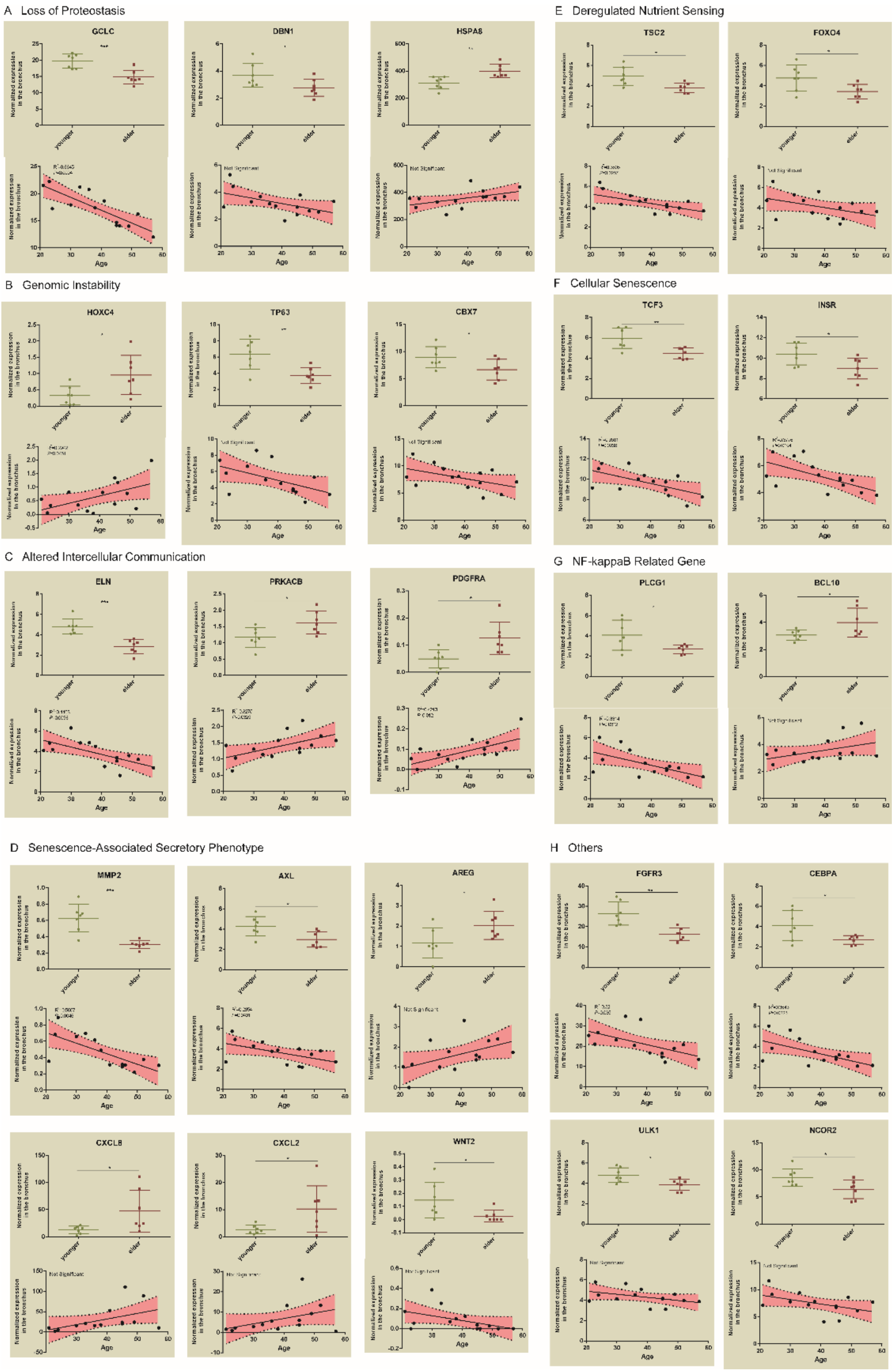

Supplementary Figure S4. GO enrichment analysis for age-related DEGs in the trachea.

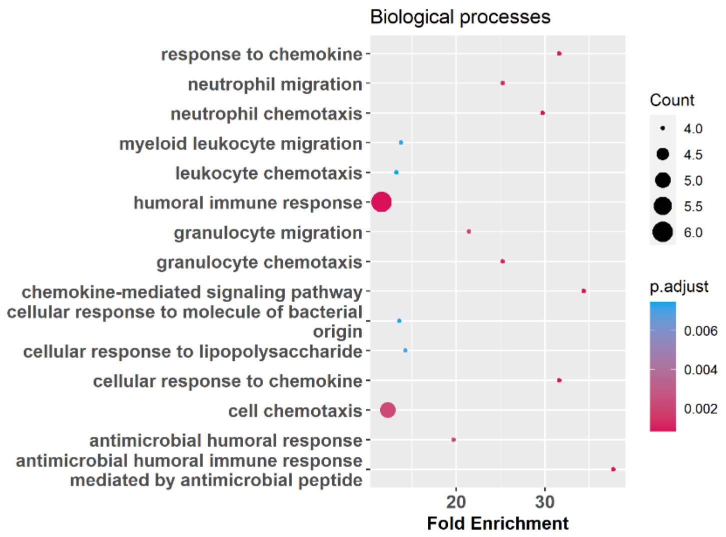

Supplementary Figure S5. GO enrichment analysis for age-related DEGs in the bronchus.

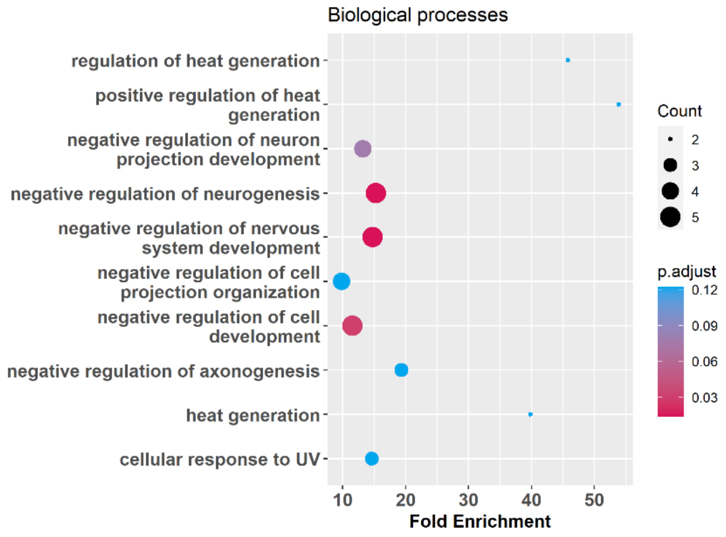

Table S1. Demographic information of the cohort.

	**Trachea**			**Bronchus**		
	Younger group	Elder group	*P* value	Younger group	Elder group	*P* value
**Subjects**	7	7		7	7	
**Gender**	female	female		female	female	

Disease states	healthy lung and airway	healthy lung and airway		healthy lung and airway	healthy lung and airway	
**Age (yrs)**						
Min-max	21-38	41-57		21-38	41-57	
Mean ±SEM	29.29 ± 2.54	47.86 ± 2.01	<0.001	29.29 ± 2.54	47.86 ± 2.01	<0.001
**Smoking**	Never	Never		Never	Never	

Notes: yrs is short for years old. SEM is short for standard error of mean. Ns is short for no significance.

Table S2. Summary of data information.

Samples	Raw Reads	Clean Reads	Clean Data(Gb)	Q30 (%)	Mapped Reads	Map Ratio(%)
Trachea-1	45,246,164	43,700,486	6.56	94.31	42,489,968	97.23
Trachea-2	45,255,560	43,708,860	6.56	93.8	41,251,190	94.38
Trachea-3	45,380,542	43,313,724	6.50	93.09	41,174,669	95.06
Trachea-4	47,314,670	45,294,016	6.79	94.33	43,626,020	96.32
Trachea-5	44,334,420	42,323,086	6.35	93.38	40,717,982	96.21
Trachea-6	46,150,008	43,385,806	6.51	93.66	41,544,541	95.76
Trachea-7	43,840,470	41,384,284	6.21	93.24	39,723,016	95.99
Trachea-8	46,521,600	45,059,468	6.76	93.97	43,759,400	97.11
Trachea-9	44,244,238	42,979,812	6.45	93.89	41,380,188	96.28
Trachea-10	46,308,720	44,824,528	6.72	94.11	42,926,222	95.77
Trachea-11	43,788,480	41,417,368	6.21	93.48	40,012,449	96.61
Trachea-12	44,590,382	42,533,528	6.38	93.31	40,628,196	95.52
Trachea-13	45,522,404	43,354,366	6.50	93.54	41,309,487	95.28
Trachea-14	42,858,826	39,936,070	5.99	92.46	37,481,145	93.85
Bronchus-1	44,384,094	42,593,396	6.39	93.67	41,378,991	97.15
Bronchus-2	45,335,710	43,774,864	6.57	93.95	40,241,370	91.93
Bronchus-3	44,158,250	41,493,366	6.22	94.13	39,775,790	95.86
Bronchus-4	44,451,794	42,695,576	6.4	93.93	41,170,491	96.43
Bronchus-5	45,508,534	43,890,032	6.58	92.89	41,969,390	95.62
Bronchus-6	44,555,412	43,042,312	6.46	94.51	41,678,972	96.83
Bronchus-7	45,536,716	43,255,162	6.49	94.18	41,738,242	96.49

Bronchus-8	45,633,384	44,245,162	6.64	94.27	42,855,202	96.86
Bronchus-9	45,112,016	43,449,960	6.52	93.84	41,998,675	96.66
Bronchus-10	45,703,156	44,503,900	6.68	95.05	42,986,767	96.59
Bronchus-11	44,542,882	42,715,296	6.41	93.78	40,958,850	95.89
Bronchus-12	46,562,886	44,912,786	6.74	93.22	42,414,294	94.44
Bronchus-13	45,243,388	43,405,220	6.51	92.95	41,445,956	95.49
Bronchus-14	45,213,652	43,421,368	6.51	93.82	41,557,439	95.71

Table S3. Cell proportions of tracheal and bronchial brushings through CIBERSORTx.

	trachea			bronchus		
	mean	SEM	n	mean	SEM	n
Suprabasal	0.0%	0.0%	14	0.0%	0.0%	14
Smooth.muscle	0.3%	0.1%	14	0.2%	0.1%	14
SMG.Goblet	2.0%	0.6%	14	2.0%	0.7%	14
Serous	0.2%	0.1%	14	0.0%	0.0%	14
Secretory	27.4%	1.5%	14	33.5%	1.5%	14
Precursor	0.0%	0.0%	14	0.0%	0.0%	14
PNEC	0.0%	0.0%	14	0.0%	0.0%	14
Plasma.cells	0.2%	0.1%	14	0.2%	0.1%	14
Pericyte	0.1%	0.1%	14	0.0%	0.0%	14
Multiciliated	40.9%	2.6%	14	44.0%	1.8%	14
Monocyte	0.8%	0.4%	14	1.0%	0.5%	14
Mast.cells	0.8%	0.2%	14	0.6%	0.1%	14
Macrophage	6.0%	2.3%	14	1.9%	0.4%	14
LT.NK	1.9%	0.3%	14	0.6%	0.1%	14
Ionocyte	0.0%	0.0%	14	0.0%	0.0%	14
Fibroblast	0.0%	0.0%	14	0.1%	0.0%	14
Endothelial	1.2%	0.3%	14	0.4%	0.1%	14
Deuterosomal	0.1%	0.1%	14	0.0%	0.0%	14
Dendritic	3.5%	0.6%	14	2.5%	0.4%	14
Cycling.Basal	6.4%	0.8%	14	5.2%	0.6%	14
Brush.cells	0.0%	0.0%	14	0.0%	0.0%	14
Basal	8.2%	1.2%	14	7.7%	0.8%	14
B.cells	0.0%	0.0%	14	0.0%	0.0%	14
AT2	0.0%	0.0%	14	0.0%	0.0%	14
AT1	0.0%	0.0%	14	0.0%	0.0%	14

Table S4. Basal cell proportions of 17 healthy lungs from GSE136831. GSE136831 is a dataset about IPF, we download the single cell RNA-seq data of 8 female and 9 male healthy lungs, and calculated the fraction of basal cells in each sample.

Subjects	Disease state	Age	Gender	Proportion of basal cells (%)
1372C	Healthy	21	female	0.50
001C	Healthy	22	male	1.00
218C	Healthy	29	male	0.04
484C	Healthy	31	male	0.09
133C	Healthy	32	female	0.04
483C	Healthy	35	male	0.20
396C	Healthy	37	female	0.20
098C	Healthy	41	female	0.30
084C	Healthy	46	male	0.20
454C	Healthy	48	female	0.09
465C	Healthy	56	male	0.20
388C	Healthy	61	male	0.20
192C	Healthy	62	female	0.02
160C	Healthy	64	male	0.04
222C	Healthy	65	male	0.06
065C	Healthy	66	female	0.10
296C	Healthy	80	female	0.10
